# GTR/Thermoplastics Blends: How Do Interfacial Interactions Govern Processing and Physico-Mechanical Properties?

**DOI:** 10.3390/ma15030841

**Published:** 2022-01-22

**Authors:** Mohammad Reza Saeb, Paulina Wiśniewska, Agnieszka Susik, Łukasz Zedler, Henri Vahabi, Xavier Colom, Javier Cañavate, Agnieszka Tercjak, Krzysztof Formela

**Affiliations:** 1 Department of Polymer Technology, Faculty of Chemistry, Gdańsk University of Technology, Gabriela Narutowicza 11/12, 80-233 Gdańsk, Poland; mohsaeb@pg.edu.pl (M.R.S.); paulina.wisniewska1@pg.edu.pl (P.W.); s171263@student.pg.edu.pl (A.S.); lukasz.zedler@pg.edu.pl (Ł.Z.); 2 Advanced Materials Center, Gdańsk University of Technology, Gabriela Narutowicza 11/12, 80-233 Gdańsk, Poland; 3 Laboratoire Matériaux Optiques, Photonique et Systèmes (LMOPS), CentraleSupélec, Université de Lorraine, F-57000 Metz, France; henri.vahabi@univ-lorraine.fr; 4 Department of Chemical Engineering, Universitat Politècnica de Catalunya Barcelona Tech, Colom 1, Terrassa, 08222 Barcelona, Spain; xavier.colom@upc.edu (X.C.); francisco.javier.canavate@upc.edu (J.C.); 5 Group ‘Materials + Technologies’ (GMT), Department of Chemical and Environmental Engineering, Faculty of Engineering, University of the Basque Country (UPV/EHU), Pza Europa 1, 20018 Donostia-San Sebastian, Spain; agnieszka.tercjaks@ehu.eus

**Keywords:** rubber recycling, ground tire rubber, thermoplastics polymer blends, compatibility, microstructure-processing-performance properties relationships

## Abstract

In this work, GTR/thermoplastics blends (in ratio 50/50 and 75/25 wt.%) were prepared by melt-compounding in an internal mixer. During research, trans-polyoctenamer rubber (TOR), ethylene-vinyl acetate copolymer (EVA), ethylene-octene copolymer (EOC), and linear low-density polyethylene (LLDPE), were used in their thermoplastic phase. Microstructure and processing-performance property interrelationships of the studied materials were investigated by: atomic force microscopy (AFM), scanning electron microscopy (SEM), rubber process analyzer (RPA), Mooney viscometer, plastometer, gas chromatography with mass spectrometry, differential scanning calorimetry (DSC), tensile tests and swelling behavior. In blends of thermoplastics with a high content of GTR (50 and 75 wt.%), the thermoplastic modifier type had a significant impact on the processing behavior and microstructure of blends. In terms of the physico-mechanical properties, the GTR/thermoplastics ratio affected elongation at break, hardness, and density, while its effect on tensile strength was negligible. DSC analysis showed that thermoplastics, as modifiers of GTR, should be considered as binders and not plasticizers, as reflected in the almost constant glass-transition temperature of the blends. RPA measurements indicated higher values of G* and η* for GTR-rich blends. SEM showed a rubber-like interfacial break, while AFM confirmed interfacial contact between GTR and thermoplastics.

## 1. Introduction

Melt-compounding is a solvent-free, low-cost and high-efficiency method for the preparation of novel polymeric materials with desired performance properties. This processing technique is also a very promising approach to rubber recycling, especially in the utilization of waste tires. Literature data have confirmed that melt-blending of thermoplastics and ground tire rubber (GTR) allows production of thermoplastics elastomers or thermoplastics/GTR blends [1,2,3], which have huge potential to be used at an industrial scale.

According to ASTM D 883 (“Standard Terminology Relating to Plastics”) thermoplastic elastomers can be defined as a “diverse family of rubber-like materials that, unlike conventional vulcanized rubbers, can be processed and recycled like thermoplastic materials”. It should be highlighted that, given suitable processing behavior, thermoplastic elastomers filled or modified by GTR should also reach the performance requirements dedicated for this class of materials. The basic parameter to evaluate the differences between thermoplastics and thermoplastic elastomers is the elongation at break, which for thermoplastic elastomers should reach at least 100% at break [4]. Another parameter for consideration is the compression set, which for thermoplastic elastomers should be lower than 50% [5]. However, it is worth mentioning that in the literature, the term “thermoplastic elastomers” is frequently incorrectly used for various thermoplastic/GTR blends which did not reach the above-highlighted requirements.

Over the last decade, several research groups have reported on different methods of preparation, formulation and modification of the properties of blends of GTR with thermoplastics. Physical blending or melt blending of GTR with thermoplastics has been considered as a very simple way to enlarge the processing window as well as to improve the physical, thermal, and mechanical properties of GTR [6,7,8].

This approach allows manipulating the interfacial adhesion between GTR particles by a physical blending of GTR with thermoplastics/elastomers or thermoplastic elastomers. In that case, there is no need for the incorporation of commercially available additives, such as curing agents, plasticizers or other processing aids. As presented in [9,10,11], the interfacial interactions level and the final performance properties of thermoplastics/GTR/elastomer systems are affected by: elastomer polarity (its compatibility with thermoplastics and GTR); melt viscosity of the thermoplastic phase; thermoplastic/GTR blend ratio and GTR particles size.

However, poor interfacial adhesion in thermoplastic filled with GTR (usually up to 50 wt.%) is related to difficulties in achieving blends containing homogeneously dispersed GTR particles into the thermoplastic phase, mainly as a consequence of a lack of compatibility between GTR and thermoplastics. Therefore, GTR devulcanization/oxidation combined with the use of suitable additives, compatibilizers, and nanofillers are recommended as the main strategies to overcome poor compatibility between thermoplastics and GTR [12,13,14,15,16].

Some recent studies claimed that thermoplastics, even at relatively low loading levels (up to 25 wt.%), can play the role of plasticizer or binder during rubber reclaiming, along with making it possible to form cross-linked rubber waste into value-added products.

Barbosa and Ambrósio [17] thermo-mechanically devulcanized natural rubber (NR) compounds in a co-rotating twin-screw extruder, varying the screw speed (350–550 min^−1^), barrel temperature (210–270 °C), and the type of thermoplastic (ethylene-vinyl acetate copolymer and polypropylene). The authors showed that using 10 wt.% in thermoplastics as auxiliary additives in rubber recycling resulted in the enhancement of mechanical properties of the prepared materials, e.g., tensile strength and elongation at break. However, better effects were observed for ethylene-vinyl acetate copolymer.

Nunes et al. [18] thermo-mechanically devulcanized GTR in the presence of 10–25 wt.% polypropylene or low-density polyethylene. The process was performed in a co-rotating twin-screw extruder at varying temperatures (220–270 °C), screw speeds (150–550 min^−1^), and throughput (5–10 kg/h). The results showed that polypropylene was more effective than low-density polyethylene in the GTR devulcanization process, promoting the formulation of sol fraction, especially at higher screw speeds.

Wang et al. [19] investigated the blends based on recycled high-density polyethylene filled with large amounts of GTR (up to 90 wt.%) compatibilized by two types of thermoplastics as a compatibilizer, namely ethylene-octene copolymer (EOC) and trans-polyoctenamer rubber (TOR). The results showed that the addition of compatibilizers enabled the production of blends with higher GTR contents and reasonable quality for automotive, packaging and construction applications.

Prut et al. [20] prepared 70/30 and 80/20 wt.% blends of GTR/thermoplastic with LLDPE, EVA with 10–14 and 24–30 wt.% of vinyl acetate, and also a random terpolymer of ethylene, vinyl acetate and maleic anhydride. The idea was to improve the mechanical properties of GTR by applying different thermoplastics, enabling the maximization of GTR processability through simple physical blending. Although 70/30 blends, showed high tensile modulus, the elongation at break was drastically decreased by the increase in GTR content. Notably, no flow was observed during melt flow rate testing for 80/20 blends. The authors also used ethylene-propylene-diene (EPDM) and boosted the interfacial adhesion over a wide composition window, reflected in large deformation of the blends [21].

In the light of the above-presented state-of-the-art trends, it can be concluded that reports about thermoplastics with high contents of GTR (above 50 wt.%) are rather limited. This is related to difficulties in processing and flowability of such high viscous systems. These limitations make it impossible to draw conclusions about the role of thermoplastics in the modification and processability of GTR. Still, there is a need for the enlargement of the processing window of GTR blends with thermoplastics using the maximal amount of GTR. This approach allows for the manufacturing of low-cost GTR/thermoplastics blends, which would have huge potential for applications in the automotive or construction industries and in building materials. Moreover, using a higher amount of GTR fits strategies of circular economy and the sustainable development of waste tire-recycling technologies.

In this work, we used EOC, TOR, EVA and LLDPE as thermoplastics to develop GTR-rich thermoplastic blends with a GTR content of 50 and 75 wt.%. Prepared materials were fully characterized by using atomic force microscopy, scanning electron microscopy, a rubber process analyzer, Mooney viscometer, plastometer, gas chromatography with mass spectrometry, differential scanning calorimetry, tensile tests and swelling behavior in order to answer the question: how do interfacial interactions govern the processing and physico-mechanical properties of GTR/thermoplastic blends?

## 2. Materials and Methods

### 2.1. Materials

Ground tire rubber (GTR) obtained from passenger and truck tires, with particle sizes of up to 0.6 mm, was received from Grupa Recykl S.A. (Śrem, Poland). The basic components of GTR are: natural rubber (NR), styrene-butadiene rubber (SBR), butadiene rubber (BR), additives (curing system, activators, plasticizers, etc.), carbon black, silica, and ash. Determined by thermogravimetric analysis, the GTR composition included: rubbers and additives (62.3 wt.%), carbon black (26.9 wt.%), silica and ash content (10.8 wt.%).

Vestenamer^®^ 8012 is a trans-polyoctenamer rubber (TOR) produced by Evonik Industries AG (Essen, Germany). According to the producer’s information, TOR can be used as a processing aid for the rubber industry, in the production of masterbatches, to increase the compatibility of rubber blends, and to simplify rubber recycling.

Escorene™ Ultra FL 00218 is an ethylene-vinyl acetate copolymer (EVA) produced by ExxonMobil Chemical (Houston, TX, USA). The vinyl acetate content in this grade is equal to 18% EVA. Producer information showed that this grade of EVA is dedicated to polymeric film production via blown or cast extrusion, lamination, etc.

Queo™ 0201FX is an ethylene-octene copolymer (EOC) produced by Borealis AG (Vienna, Austria). According to the technical data sheet, this grade is intended for use as a primary blend partner in high-performance seal layers. This additive is designed to offer improved control of the coefficient of friction of coextruded blown films.

LLDPE MG500026 is a high-flow linear low-density polyethylene (LLDPE) produced by Saudi Basic Industries Corporation (Riyadh, Saudi Arabia). This grade of LLDPE is recommended for injection molding masterbatch, where a high filler acceptance is required, combined with a good flow.

**Figure 1:**
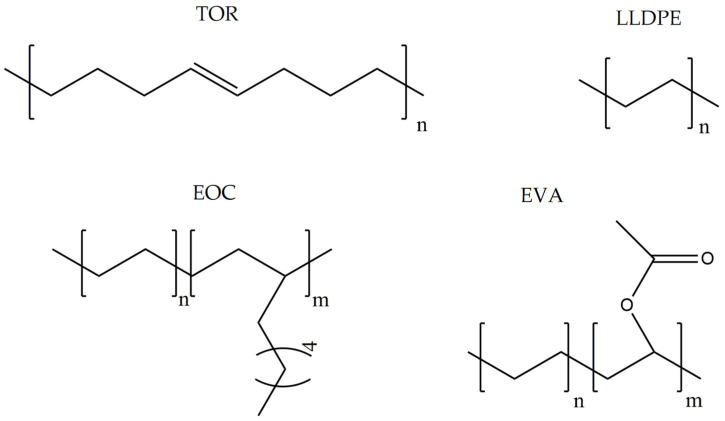
Chemical structures of used thermoplastics modifiers.

Characteristics of components used in this study are presented in Table 1, while their structures are shown in Figure 1. All components are commercially available and were used as received.

### 2.2. Sample Preparation

GTR was blended with 50 wt.% or 25 wt.% of thermoplastic polymer. The samples were prepared in the Brabender^®^ internal mixer (type GMF 106/2) (Brabender GmbH & Co. KG, Duisburg, Germany) with a chamber volume of approx. 55 cm^3^, equipped with roller-type rotors. The temperature and rotor speed during mixing were 180 °C and 120 rpm, respectively. The mixing time for all blends was 8 min. The thermoplastic polymer was melted for 1 min, then GTR was added and the mixing procedure was continued for the next 7 min. Subsequently, the prepared blends were compressed into 2 mm-thick tiles or dumbbell-shaped samples via compression molding under a pressure of 4.9 MPa at 180 °C for 1 min and then at room temperature for 5 min. The sample composition and coding are summarized in Table 2.

### 2.3. Methodology

Melt mass-flow rate (MFR) and melt volume-flow rate (MVR) of the GTR/thermoplastic blends were investigated using a Zwick mFlow plastometer (Ulm, Germany) according to ISO 1133 (combined methods A and B) at 190 °C, with a load of 5 kg.

Mooney viscosity of the rubber compounds was measured at 100 °C using a Mooney viscometer MV2000 (Alpha Technologies, Akron, OH, USA) according to ISO 289-1.

Premier RPA Enhanced (Alpha Technologies, Akron, OH, USA) was used to determine the processing parameters of the GTR/thermoplastic blends. The samples were cut from compression-molded 2 mm tiles and around 4.5 g was placed into the measurement chamber. Measurements were performed at 0.1 Hz and at two temperatures (100 °C and 190 °C) at a strain of 150% and 300%. The stabilization time was equal to 1 min and test time was 8 min.

The tensile strength and elongation at break were estimated in accordance with ISO 37. Tensile tests were performed on a Zwick Z020 apparatus (Ulm, Germany) at a constant speed of 300 mm/min. Shore hardness type A was estimated using a Zwick 3130 durometer (Ulm, Germany) in accordance with ISO 868. At least 5 measurements per sample were performed.

The density of samples was measured based on the Archimedes method, as described in ISO 1183. Accordingly, all measurements were carried out at room temperature in methanol medium. At least 3 measurements were performed per sample.

The swelling degree of the blends (approx. 0.2 g samples) after 72 h was determined by equilibrium swelling in toluene (at room temperature). The swelling degree was calculated in accordance with the Equation (1):

(1) $$Q=\frac{m_{t}-m_{0}}{m_{0}}\times 100\%$$

where: *Q*—swelling degree, %; *m_t_*—mass of the sample swollen after 72 h, g; *m*_0_—an initial mass of sample, g.

The sol fraction was determined on the basis of the mass difference between the initial sample and the dried sample after extraction according to Equation (2):

(2) $$F_{sol}=\frac{m_{0}-m_{k}}{m_{0}}\times 100\%$$

where: *F_sol_*—the content of sol fraction, %; *m*_0_—an initial mass of the sample, g; and *m_k_*—a mass of the dried sample after extraction, g.

A sampling of volatile organic compounds (VOCs) emitted to the atmosphere during the melt-compounding of GTR/thermoplastic blends was performed using a passive sampling technique with a Radiello^®^ diffusing sampling system (Fondazione Salvatore Maugeri, Padova, Italy). Radiello^®^ diffusive passive samplers are based on three main components: a microporous polyethylene diffusion membrane, a plastic tripod dedicated for installation of the sampler, and a cylindrical steel net filled with graphitized charcoal Carbograph 4 as a sorption bed. A sampling of VOCs directly from the mixing chamber was carried out for 8 min via the Radiello^®^ system. VOCs collected on the Radiello^®^ diffusive passive samplers were liberated using the thermal desorption technique (Unity v.2, Markes International Ltd., Pontyclun, UK), connected with a gas chromatographer (Agilent Technologies 6890) combined with a mass spectrometer (5873 Network Mass Selective Detector, Agilent Technologies, Santa Clara, CA, USA). More detailed information about this methodology is presented in works [22,23,24].

The morphology of the surface created by breaking the samples in the tensile test at a speed of 500 mm/min was observed with a JEOL 5610 scanning electron microscope (Tokyo, Japan) with a low-vacuum secondary electron detector. During the analysis, the electron beam accelerating voltage was 10 kV. Before measurement, the samples were covered with a fine gold layer in order to increase their conductivity in the vacuum chamber.

Atomic force microscopy (AFM) was carried out for a deeper study of the morphology as well as the interface between both the GTR and thermoplastics. AFM images were obtained by using an AFM ICON Nanoscope V from Bruker (Billerica, MA, USA), equipped with an integrated silicon tip/cantilever (TESP-V2, 125 m in length with ca. 300 kHz resonant frequency) and working in the tapping mode. Typical scan rates during recording were 0.7 to 1 line/s. All investigated blends were cut using a diamond knife to study their transversal cross-section. The roughness of the surfaces was determined as the roughness average using AFM height images (20 × 20 μm).

The thermal analysis of the investigated GTR/thermoplastic blends was carried out by differential scanning calorimetry (DSC) using Mettler Toledo DSC3+. The 5–10 mg of each blend sealed in an aluminum pan was heated, cooled, and heated again from −80 °C to 200 °C at a heating/cooling rate of 10 °C/min under a nitrogen atmosphere. The date was taken for the 2nd heating to avoid thermal history.

## 3. Results and Discussion

### 3.1. Processing Characteristics of GTR/Thermoplastics Blends

Table 2 summarizes the values of MFR/MVR, Mooney viscosity, and RPA measurements for the studied samples. The first two (MFR/MVR and Mooney viscosity) can be taken as standardized tests showing the potential of the flow behavior of blends, considering the fact that the different linearity and structure of thermoplastics (see Table 1 and Figure 1) can play a key role in the processing and properties of the resulting blends. RPA gives a very unique signature of dynamic processing and fluidity of the highly viscous systems, to be used in the analysis of real-scale processing of GTR-rich blends. Two compositions of GTR/thermoplastic blends, namely 50/50 and 75/25 wt.% could also reflect the processability and possibility of maximal loading of GTR. In terms of the measurements, the Mooney viscosity, MFR/MVR, and RPA tests conducted on the blend samples were performed at 100 °C and 190 °C, respectively, such that GTR blends with thermoplastics could be compared from a processability point of view with neat rubbers/thermoplastic elastomers (Mooney viscosity) and thermoplastics/thermoplastic elastomers (MFR/MVR). The values of Mooney viscosity measured at 100 °C for 50/50 blends of GTR/TOR and GTR/EVA were 11.7 and 42.8, respectively. On the other hand, for 50/50 GTR/EOC and GTR/LLDPE blends, no data was collected. Since Mooney viscosity reflects the resistance of an uncured rubber compound [25], the application of this method for partially cross-linked polymers or polymeric materials filled with GTR might be limited. The results showed that the presence of thermoplastics with softening and melting temperatures close to 100 °C (EOC, LLDPE) affects the rubber-like character and elasticity of modified GTR, which resulted in overloading of the torque during Mooney viscosity measurements.

As presented in Table 1, the melting temperatures of the thermoplastics in the same order were 54, 87, 95, and 122–124 °C, TOR was well above its melting temperature and EVA, to some extent, had a good fluidity. By contrast, EOC and LLDPE could be assumed to be almost solid or pasty phases with no flow when blended with GTR. The softening points of EVA, EOC, and LLDPE are 62, 80, and 88 °C, while TOR melts sharply. These observations are quite expectedly in agreement with the MFI values of 50/50 blends. To gain an understanding of fluidity in the system, an MFR/MVR test on 50/50 blends was conducted at 190 °C, but under a 5 kg static force. Glancing at the MFR/MVR values of 50/50 systems gives an idea of how thermoplastics added to GTR at 50 wt.% governed the properties.

Evidently, LLDPE, with a dominant linear backbone and low molecular weight (MFR equal to 50 g/10 min, 190 °C, and 2.16 kg), provided GTR with good fluidity, such that the MFR of 50/50 GTR/LLDPE was 28.4 g/10 min, while those of the 50/50 systems of GTR/TOR, GTR/EVA, and GTR/EOC were 11.45, 2.04, and 1.01 g/10 min, respectively. These results once again emphasize the better processability of GTR/TOR systems compared to GTR/EVA or GTR/EOC. In other words, the presence of methyl acetate repeating units in EVA or octene segments in the EOC microstructure with a greater degree of freedom for movement, lower melting point and softening point compared to LLDPE, is the reason for such differences. Thus, thermoplastic nature and microstructures play a key role in the fluidity of 50/50 GTR-based blends. The conclusion concerning 50/50 systems is that thermoplastics under such circumstances play the role of a plasticizer, which will be later discussed in terms of microstructure and properties. For 75/25 GTR-based blends, however, completely different behavior is observed, which can be explained on account of processing and molecular parameters. Higher values of Mooney viscosity for 75/27 GTR/TOR (36.8) and GTR/EVA (73.6) compared to the corresponding 50/50 systems are characteristic of higher resistance of blends against the rotation of the disk inside the cylinder of the apparatus, which can be ascribed to higher content of GTR in the blend. However, again for GTR/EOC and GTR/LLDPE, no data was collected. When comparing MFR values of 75/25 blends, only GTR/LLDPE gives some data; however, the standard deviation for this sample is higher compared to 50/50 systems, which might be related to the partial decomposition of GTR during the test. It should be emphasized that even in the TGA test in the absence of shear, the degradation of GTR is considerable at around 200 °C [26]. On the other hand, due to the higher affinity of other thermoplastics to GTR, particularly TOR [27], a kind of network formation inside the tube of the capillary might be responsible for partial revulcanization of GTR—the reason why the aforementioned blends show no flow during MFR testing.

For samples undergoing rubber process analyzer (RPA) testing at 100 °C or 190 °C, the dynamic mechanical behavior could be interpreted and compared with the outcomes of the Mooney and MFI analyses. According to the results at 100 °C, complex shear modulus (G*, kPa) and complex shear viscosity (η*, Pa·s) were obtained as a function of oscillation strain (%), and values at 150 and 300% were reported. Regardless of blend composition, the GTR/LLDPE systems were in chaos, possibly because of a lack of softening due to the high melting point of LLDPE (≈123 °C, which is well above the test temperature, 100 °C). For other systems, the higher the strain rate, the lower both G* and η*, possibly because of the destruction of aggregate-aggregate rubber networks formed during blending. The values of G* at 150 and 300% can be roughly correlated with the Mooney viscosity fluctuation trend observed, since both criteria reveal the resistance of the rubber compound against flow. Overall, somewhat similar behavior was observed for 50/50 blends, such that TOR was well below its melting temperature during RPA measurement at 100 °C, which may be the reason for significantly lower G* and η* values for GTR/TOR at both 150 and 300% strain, compared to GTR/EVA and GTR/EOC samples. Moreover, EOC and EVA show more affinity to GTR; therefore, a thicker interphase region with GTR particles might be formed. For 75/25 GTR-dominant blends, G* and η* variations for different types of blends and at both strain rates follow a similar trend, but values are obviously higher because GTR is makes up the majority proportion in the blend. In fact, a rubber network formed in 75/25 systems needs much more torque to be destroyed; therefore, higher values of G* and η* are justifiable. The point to be highlighted is that RPA results show considerable deviation, probably because of the sensitivity of testing to phase morphology and the lack of adequate interfacial interaction in a system free of any compatibilizer or curing agent. In the other words, although revulcanization of GTR with thermoplastics is a possibility, the interfacial adhesion does not demonstrate adequate resistance in dynamic tests. As a result of the destruction of such physical interactions or the limited number of chemical bonds which could be formed at 100 °C, the vivid character of GTR blends was unveiled in dynamic analyses. Analyses at 190 °C provided us with more insights about the interfacial interaction status in the system. Although trends in the variation of G* and η* at 190 °C are somewhat similar to those at 100 °C, the absolute values and deviations are obviously lower at 190 °C. Moreover, values of G* and η* at 190 °C are considerably higher for 75/25 systems with respect to 50/50 ones, signifying a more rubber-like blend because of GTR making up the majority proportion. Conspicuously, RPA of GTR/LLDPE blends at 190 °C (particularly 75/25 GTR/LLDPE blend) makes sense, because this temperature is able to soften LLDPE to some extent. All in all, at the higher temperature of 190 °C, all the thermoplastics are above their softening and melting point, which gives a real picture of the processability of blends and interfacial interactions in the systems.

### 3.2. Volatile Organic Compound Emission Profile during GTR/Thermoplastics Blends Preparation

**Figure 2:**
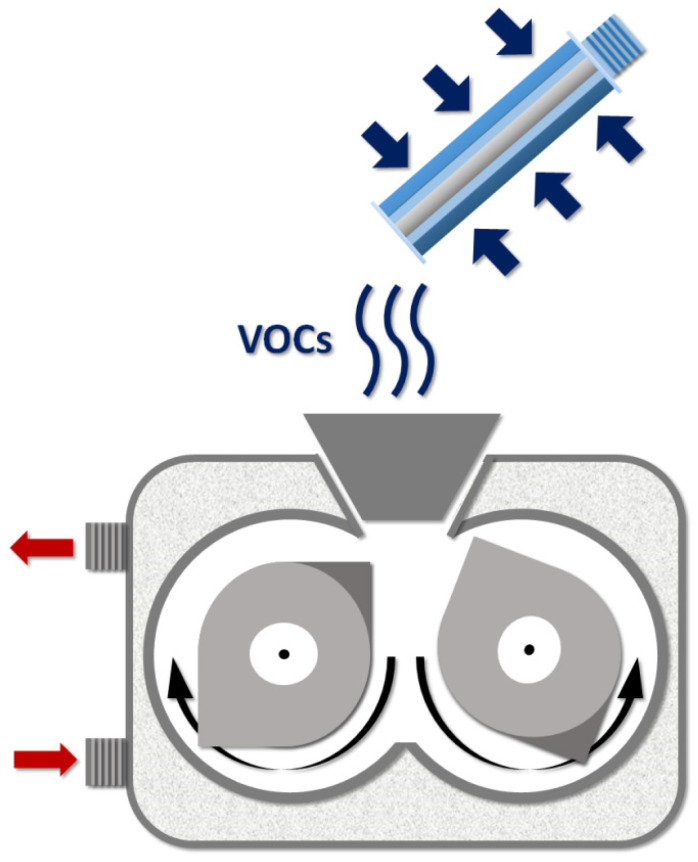
Scheme of VOCs collection during sample preparation by using Radiello^®^ diffusive sample system.

To determine the emission of volatile organic compounds released during GTR/thermoplastics blends preparation, the Radiello^®^ diffusive sample system was used. The scheme of the VOCs collection is presented in Figure 2.

Table 3 summarizes the chemical structures of volatile organic compounds emitted from GTR/thermoplastics blends, determined by GC-MS analysis. It can be seen that the identified volatile compounds are mostly degradation products of GTR, which can be divided into two basic groups: (i) natural rubber degradation products (cyclooctane; decane; limonene; undecane; dodecane; tridecane; tetradecane); and (ii) styrene-butadiene rubber degradation products (benzene; toluene; ethylbenzene; xylene; styrene; benzaldehyde; α-methylstyrene). It should be noted that sulfur compounds or accelerator residue in the detected structures were not observed, which indicates that the main reclaiming mechanism during GTR/thermoplastics blends preparation is scission of the main chains of rubber macromolecules. The presence of limonene—the product of natural rubber degradation (one of the main components of GTR)—as a marker confirms this assumption.

### 3.3. Physico-Mechanical Properties of GTR/Thermoplastics Blends

Typically, interfacial adhesion between polymers in a binary blend is the controlling parameter affecting and determining the ultimate properties, particularly the mechanical characteristics, of blends. A good interfacial bonding is typically characteristic of compatibilized polymer blends, where higher mechanical properties are achieved. However, sometimes composition should be considered as the game-changer, particularly when the secondary phase plays the role of binder or toughening agent. There are always serious difficulties accompanied by the interpretation of the mechanical properties of systems comprising rubber particles because of the possibility of the encapsulation or aggregation of rubber particles, depending on their surface chemistry, size, and composition. In the absence of an external compatibilizer, physical interactions or physical networks formed via hydrogen bonding or van der Waals forces are the controlling factors. The mechanical properties of the samples prepared in this work were evaluated in terms of the composition of GTR in the blends. As shown in Table 4, the tensile strength, elongation at break, and hardness of GTR/thermoplastic blends prepared in wt.% composition of 50/50 and 75/25 GTR/X, with X denoting TOR, EVA, EOC, and LLDPE, were measured, where two general behaviors were more or less detected depending on composition.

**Figure 3:**
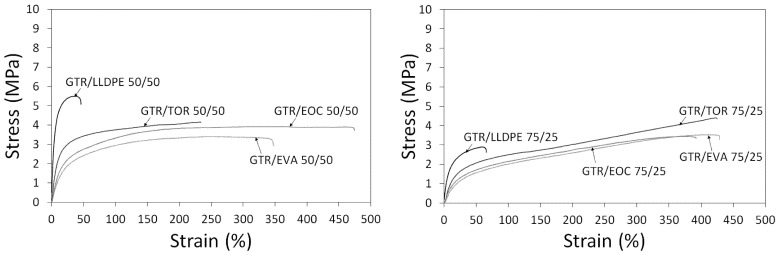
Stress-strain curves of studied GTR/thermoplastics blends as function of ratio: 50/50 (**left side**) and 75/25 (**right side**).

For 50/50 wt.% blends, the elongation at break of the GTR-based blend was increased from ca. 290% to 330% when TOR was replaced by EVA. Likewise, an increase in elongation from 330% to 457% was observed when EVA was replaced by EOC. However, blending LLDPE with GTR in a 50/50 wt.% ratio drastically decreased elongation to around 40%. For 75/25 wt.% GTR/X blends, an almost similar behavior was observed, such that 440, 440, 380, and 66% elongation at break values were observed by addition of TOR, EVA, EOC, and LLDPE as thermoplastics in lower amounts than GTR. This can be simply ascribed to the polarity of TOR, EVA, and EOC compared to LLDPE. Of note, the standard deviation of systems containing TOR, EVA, and EOC was changed more sensibly, in opposition to those of LLDPE-incorporated systems. It is also important to highlight that a lower amount of LLDPE in the 75/25 system was more efficient to have a material with higher elongation. The tensile strength values of 50/50 systems of GTR/TOR, GTR/EVA, GTR/EOC, and GTR/LLDPE are 4.4, 3.4, 3.9, and 5.4 MPa, respectively. In the same order, values of the tensile strength for 75/25 wt.% systems were 4.4, 3.4, 3.4, and 2.9 MPa. The higher value of tensile strength in the case of the 50/50 GTR/LLDPE system is again a signature of the fact that LLDPE plays the role of processing aid for GTR, in contrast to the other thermoplastics with dominant roles in interfacial interaction reinforcement. When the GTR content in thermoplastics increases, the tensile strength is expectedly decreased, but elongation at break is increased because of the dominance of rubber-like behavior, regardless of the GTR/thermoplastic composition. This can be simply understood from the stress-strain behavior of the samples compared in Figure 3. GTR/LLDPE samples have a vividly higher modulus compared to other samples, irrespective of the composition. There are some reports highlighting the fact that GTR contribution up to 65 wt.% leads to partial aggregation in the HDPE matrix, where small voids form between the GTR and thermoplastic HDPE and play the role of stress concentration zones, leading to a drop in elongation at break [35]. On the other hand, in other systems (particularly in 75/25 composition), GTR determines the deformation behavior in tensile testing, such that the stress-strain curves of almost all samples (except for GTR/LLDPE) are overlaid on each other. When the composition of the thermoplastic changes, the difference comes from different affinities among thermoplastics to GTR, where a 50/50 GTR/EOC blend (as discussed in the processing section) showed greater deformation behavior compared to GTR/TOR and GTR/EVA. Thus, the composition and thermoplastic type coincidentally control the mechanical behavior of a given GTR/thermoplastic system.

Overall, the formation of GTR domains surrounded by thermoplastic in the case of 50/50 systems was highly likely, whereas in levels above 50 wt.%, GTR played the role of dominant phase as it controlled the flow and properties such as melt strength and flow behavior of thermoplastics (see Table 2). In terms of hardness (Shore A), 50/50 wt.% GTR/TOR, GTR/EVA, GTR/EOC, and GTR/LLDPE systems had values of 82, 74, 80, and 92, respectively. We can see that GTR/LLDPE system still looks different. On the other hand, values for 75/25 systems were 69, 65, 69, and 79, in the same order, which demonstrate the dependency of hardness on the composition. Due to the formation of GTR/thermoplastic core/shell particles, a thicker thermoplastic layer around GTR is more likely in 50/50 blends, so the corresponding values of 50/50 wt.% systems are obviously higher than those of 75/25 blends. Other researchers have mentioned that even compatibilization of GTR/LLDPE (or HDPE) blends with the composition of GTR reaching up to 70 wt.% are not representative of a significant improvement in mechanical properties [36]. Irradiation of compatibilized blends of GTR with EVA (50/50 blend) also makes the blend brittle, even though the dispersion of rubber domains in the EVA matrix can be improved [37].

The density of blends was measured at temperatures of 20 and 190 °C (see Table 4). Except for the GTR/LLDPE system, the density of 75/25 systems could not be measured because of a lack of flow from the apparatus (Table 2). The higher density of 75/25 systems at 20 °C would be a reason for the formation of a denser colony or network of rubber, which is logical in view of the higher density of GTR (≈1.178 g/cm^3^) compared to that of thermoplastics (0.902–0.940 g/cm^3^) (please see Table 1). Moreover, at 190 °C the density values for 50/50 blends are lower than those at 20 °C because of greater fluidity and packing in the system. Measurements on swelling were indicative of a lack of data for the GTR/TOR system, because of the dissolution of TOR in toluene. Moreover, swelling measurements revealed no meaningful difference between the solvent content adsorbed with GTR/EVA in 50/50 and 75/25 compositions (≈170 and 183%, respectively), which has been confirmed by sol fraction (≈11 and 12.8%, respectively). This could be characteristic the formation of a rubber-like network along with a core/shell structure having EVA as a shell-forming component. As postulated beforehand, 50/50 wt.% blends in which the thermoplastic phase is in the majority make a clearer difference between the potentials of thermoplastics in interaction with the GTR. In this sense, GTR/EOC and GTR/LLDPE revealed 80 and 66% swelling, far less than the 170% obtained for GTR/EVA. In 75/25 blends, GTR formed the majority of the mixture, such that the aforementioned systems showed a high potential for swelling, with values of ≈120 and 109%. The amount of sol formed in the system followed a similar trend, which means that GTR content determines the swelling behavior. Thus, once GTR forms the majority of the mixture, the type of thermoplastic is not a determining factor. On the other hand, core/shell particles with a thicker thermoplastic layer around GTR particles can be easily dissolved in toluene. Notably, the difference between swelling content in the GTR/LLDPE system significantly changes from 50/50 to 75/25 compositions, almost twice. This means that LLDPE can assist GTR in receiving more solvent in the 75/25 system, playing the role of a binder rather than a softener in the 50/50 blend.

For a better understanding of the role of thermoplastics in the performance properties of GTR/thermoplastic blends, the comparison of results published by independent research groups are summarized in Table 5. As can be noticed, in all presented compositions, tensile strength varied in the range of 1.6–7 MPa, while elongation at break varied from 10–140%. In this study, GTR/thermoplastic blends were characterized by tensile strength in the range of 2.9–5.4 MPa and elongation at break in the range of 41–611%. This comparison shows similarities in the values of the tensile strength of GTR/thermoplastic blends, while the processing conditions and kind of thermoplastics have significant impacts on elongation at break (the crucial parameter used for the classification of materials as thermoplastic elastomers).

### 3.4. Microstructure of GTR/Thermoplastics Blends

**Figure 4:**
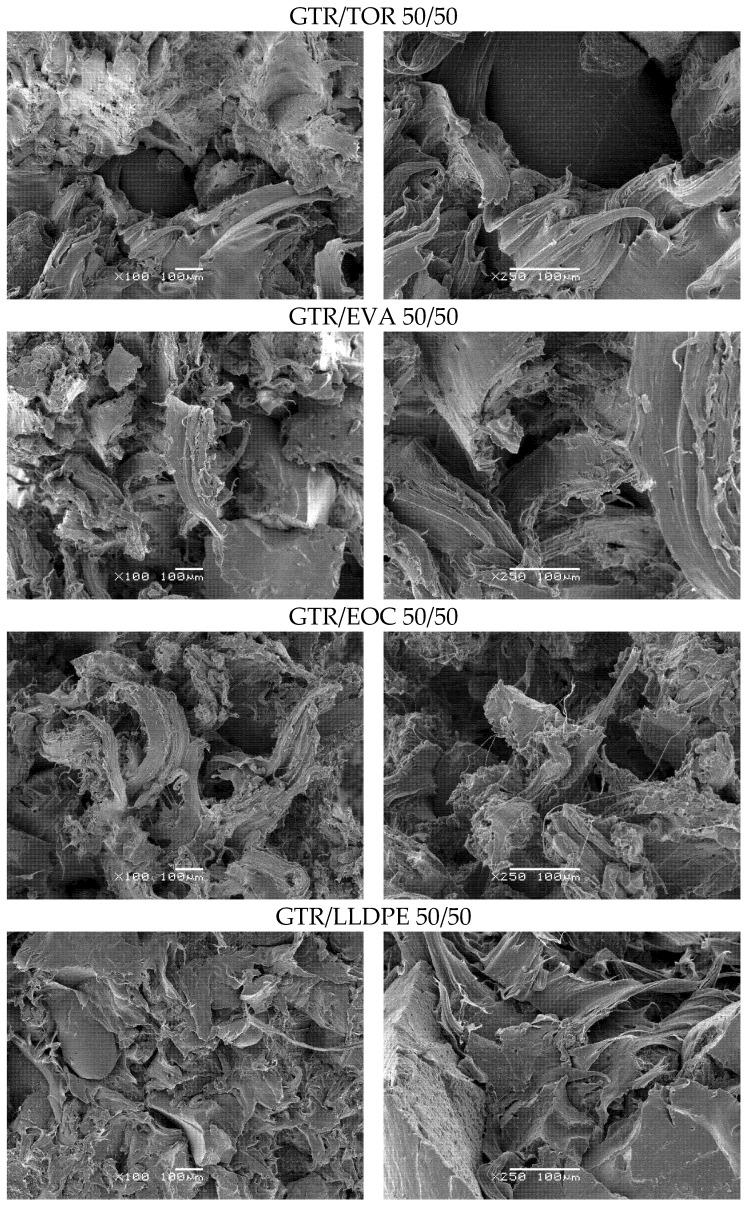
SEM images of GTR/thermoplastics blends in a ratio of 50/50 wt.% (**left side**—magnification ×100, **right side**—magnification ×250).

Morphological analysis conducted on the fracture surface of blends gives useful information about the status of interfacial interactions in the system. As presented in Figure 4, for 50/50 wt.% blends, except for GTR/LLDPE, the systems show a ductile breakup, particularly the GTR/EOC system with an elongated rubber phase at fracture surface. This observation strongly supports the results on elongation at break in 50/50 systems (see Table 4), where GTR/EOC with a high affinity to GTR revealed 457% as the highest value of elongation at breaking point. Bearing in mind the possibility of the formation of a thermoplastic layer around GTR in 50/50 blends, one would consider a thicker shell layer due to the formation of EOC around GTR domains.

**Figure 5:**
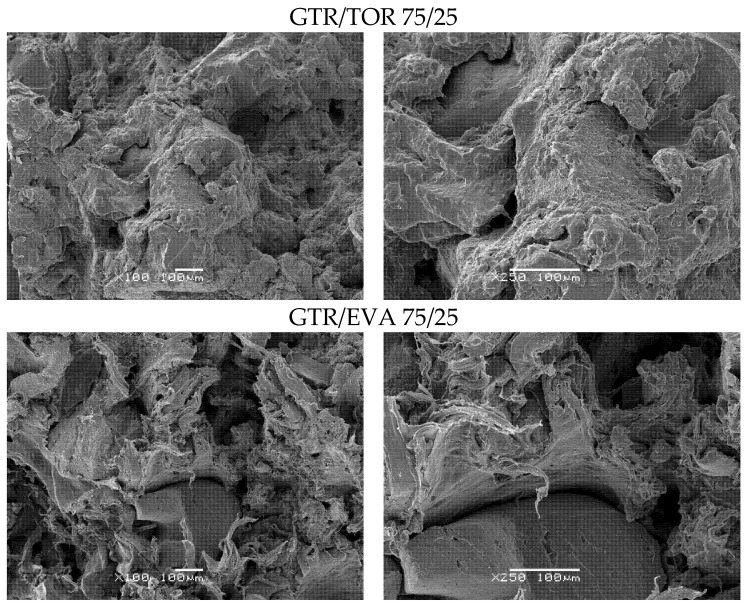

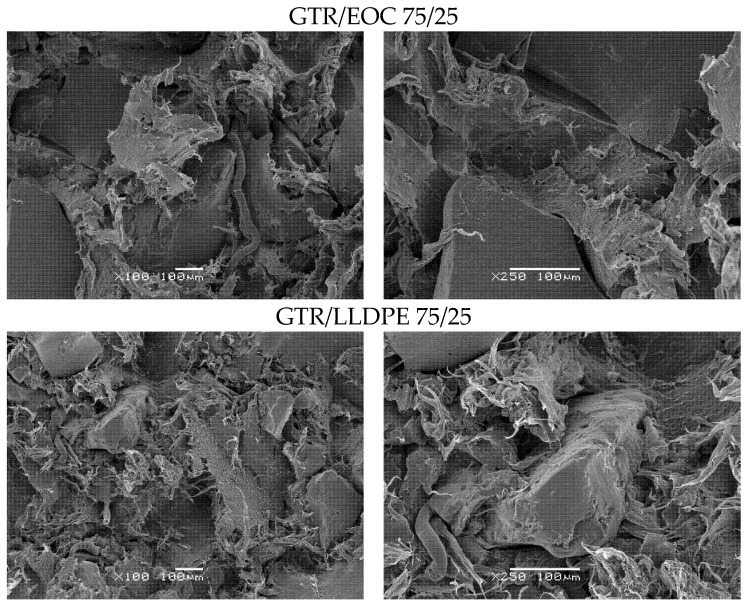
SEM images of GTR/thermoplastics blends in ratio 75/25 wt.% (**left side**—magnification ×100, **right side**—magnification ×250).

For 50/50 GTR/LLDPE, a very low value of elongation of 41% can be simply corroborated considering the fragile surface of the sample observed in Figure 4. Values of 293 and 329% for elongation at break of 50/50 GTR/TOC and GTR/EVA blends are also in agreement with the shape of deformed particles at the fracture surfaces in the stress-strain curves presented in Figure 3. The images on the right in Figure 4 and Figure 5 provide a closer view of the interaction state in the fractured surfaces.

As presented in Figure 5, for 75/25 wt.% GTR/thermoplastics blends, a more uniform morphology was observed for all of them. Compared to 50/50 systems, a more fibrillar fracture surface can be observed in 75/25 blends, which is characteristic of rubber-like behavior. For GTR/TOR system, a uniform fracture surface can be seen (Figure 4), which is in accordance with a very high elongation at break of 440% obtained for this sample (Table 4). The GTR/EVA 75/25 system also shows a uniform fracture surface with ductile fracture, ascribed to 436% breakup elongation. For 50/50 blends, the dispersion of reclaimed rubber within LLDPE and the tensile strength of the order of results of this survey have already been reported [40]. Moreover, it should be highlighted that blends based on a rubber-like polyolefin to some extent made the interface strong, leading to more uniformity and dispersion as a result of the formation of the core-shell droplets with thermoplastics and GTR as core-forming components in a relatively complex system, which was described by Li et al. [41]. The authors also noticed that polarity plays a key role in achieving appropriate adhesion between phases. The results demonstrated that the addition of non-polar EOC to a high-density polyethylene/ground tire rubber blend enhances encapsulation of GTR by EOC phase, while for polar EVA, phase separation between GTR and EVA was observed, which determines mechanical strength [41]. However, in the presented example, the impact of elastomer polarity on its compatibility and interfacial adhesion with high-density polyethylene should be also considered.

**Figure 6:**
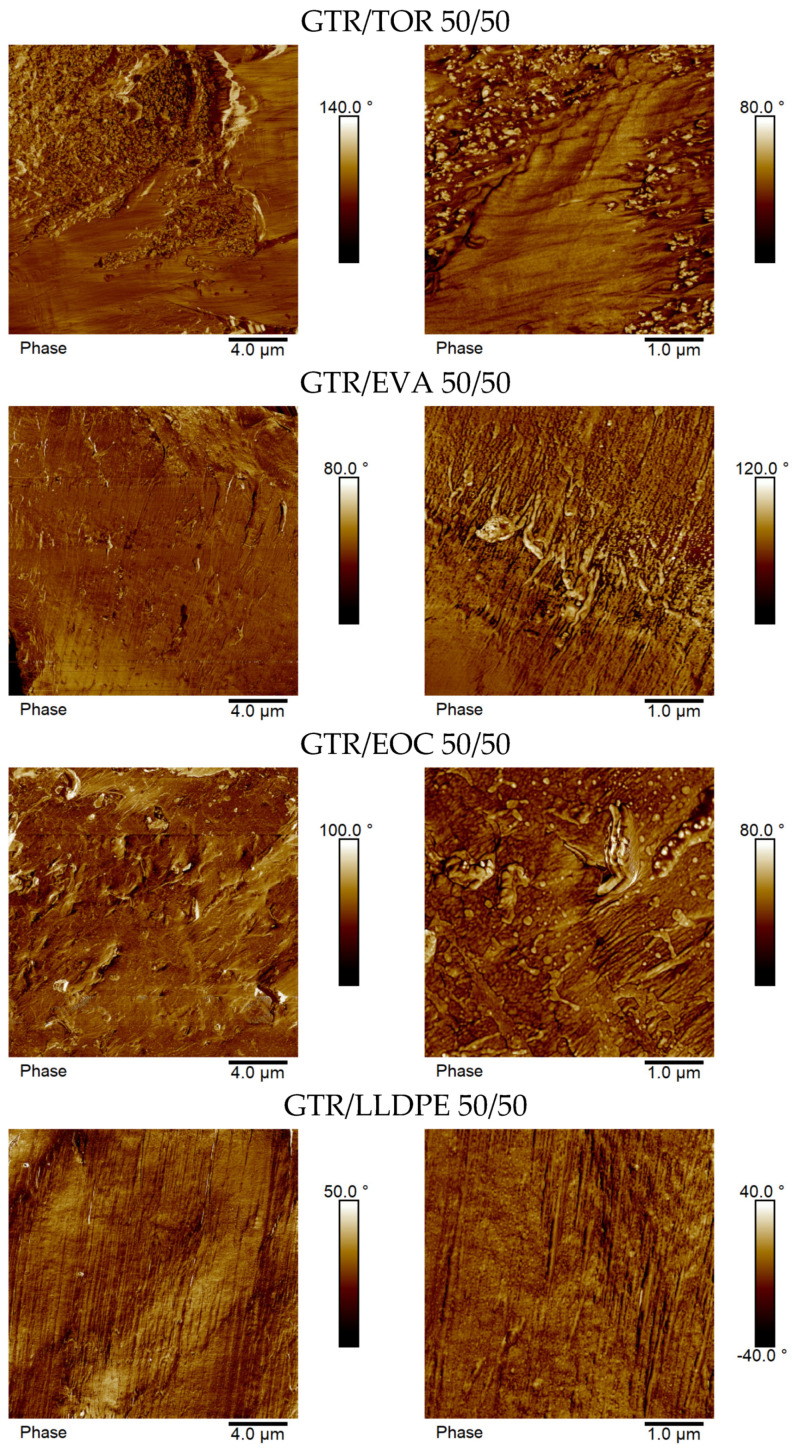
Typical cross-section AFM phase images (20 × 20 μm (**left**) and 5 × 5 μm (**right**)) of GTR/thermoplastic blends with ratio 50/50 wt.%.

**Figure 7:**
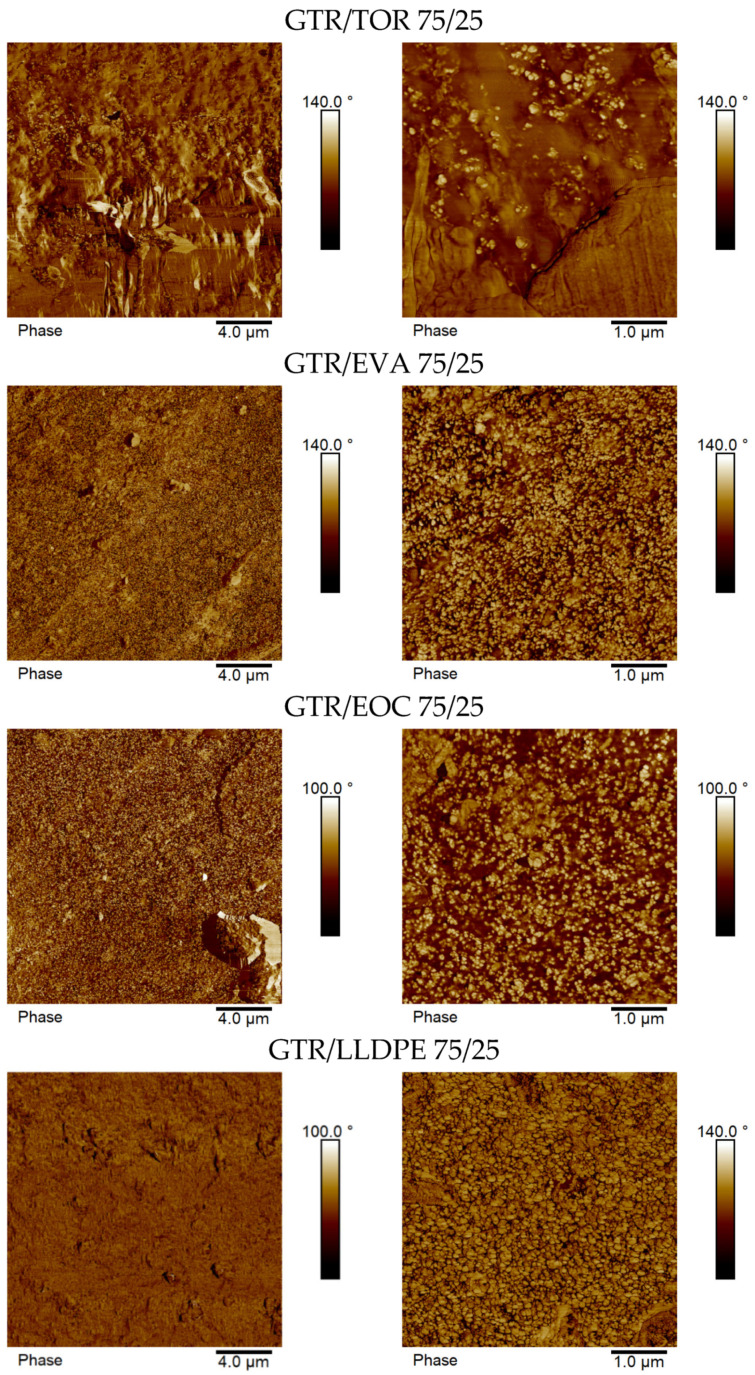
Typical cross-section AFM phase images (20 × 20 μm (**left**) and 5 × 5 μm (**right**)) of GTR/thermoplastic blends with ratio 75/25 wt.%.

Typical cross-section AFM phase images of GTR/thermoplastic blends in two different sizes (20 × 20 μm and 5 × 5 μm) are shown in Figure 6 for the sample with a ratio of 50/50 wt.% and in Figure 7 for the sample with a ratio of 75/25 wt.%. For all investigated GTR/thermoplastic blends, taking into account the macroscale size of the separated blend components (see corresponding SEM images in Figure 4 and Figure 5), the AFM technique was only used for the analysis of the compatibility between separated phases. As can be clearly extracted from the AFM phase images for GTR/thermoplastic blends with a ratio of 50/50 wt.%, none of the investigated blends showed any gap between the components (roughness of the surface calculated from the AFM height images (20 × 20 μm) equal to 380 nm, 350 nm, 160 nm, and 150 nm for GTR/thermoplastic blends with TOR, EVA, EOC, and LLPPE, respectively). This suggests an appropriate connection between the GTR-rich phase and the thermoplastic-rich phase, whatever the type of thermoplastic. This good connection between the blend components is obviously visible in 5 × 5 μm AFM phase images. Thus, although there is no external reactive compatibilizer in the system, the compatibility between blend components in 50/50 wt.% seems adequate. Nevertheless, different roughness quantities corroborate the role of the thermoplastic type, which has been reflected in the mechanical properties (Figure 3).

Moreover, AFM phase images showed that, in the case of GTR/thermoplastic blends with a ratio of 75/25 wt.%, the used thermoplastics are embedded in the GTR-rich phase, which is especially visible for GTR/thermoplastic blends with EVA, EOC, and LLPPE. As in the case of GTR/thermoplastic blends with a ratio of 50/50 wt.%, the investigated ultramicrotomy cut surface is smooth, without gaps between the blend components (roughness of the surface calculated from the AFM height images (20 × 20 μm) was equal to 370 nm, 390 nm, 240 nm, and 93 nm for GTR/thermoplastic blends with TOR, EVA, EOC, and LLPPE, respectively). It can be seen that the largest value is about four times the smallest one dedicated to the GTR/LLDPE system. Such a difference was, however, around 2.5 times for 50/50 wt.% blends, a characteristic of the dominance of the GTR role. This is also in good agreement with the mechanical properties of 75/25 blends with similar tensile strength, except for the GTR/LLDPE blend (see Figure 3).

### 3.5. Thermal Properties of GTR/Thermoplastics Blends

DSC analysis gave useful information about the thermal behavior of GTR blends with thermoplastics. It goes without saying that the melting temperature (T_m_) of the blend belongs to a thermoplastic component. It can be seen from Table 6 that GTR blends show an almost similar T_m_ not obviously affected by GTR, i.e., almost T_m_ of neat thermoplastics.

However, the amount of fusion heat (ΔH_m_) increases per weight of thermoplastic in the blend, so that it doubles by increasing the thermoplastic content from 25 to 50 wt.%. The most important outcome of DSC is that T_g_ has remained almost constant regardless of thermoplastic type or content. The obtained values are comparable with the glass transition temperature of GTR measured by Scuracchio et. al. [42], which was 59.7 °C. It means that the GTR phase determines the T_g_ of the blend, or that thermoplastics are almost binders rather than being the plasticizing agent for GTR. For GTR/HDPE systems, a similar trend was observed up to 65 wt.% of GTR, but no information was provided about the T_g_ of the blend to explicitly discuss the plasticizing effect of HDPE towards GTR [35].

## 4. Conclusions

In this work, we attempted to examine the highly loaded GTR blends with thermoplastics. Based on the literature on thermoplastic/GTR blends, we chose TOR, EOC, LLDPE and EVA and prepared their blends with GTR at 50/50 and 75/25 wt.% Analyses demonstrated that LLDPE behaves differently from both processing and property viewpoints with respect to other thermoplastics, such that the highest melting temperature and linear architecture show the lowest affinity to GTR. Higher values of Mooney viscosity for 75/25 GTR/TOR and GTR/EVA compared to corresponding 50/50 systems were indicative of rubber network formation in 75/25 systems. The processability of thermoplastic/GTR blends at 100 and 190 °C was dependent on the melting temperature, so that EOC and LLDPE were not molten at 100 °C, while EVA and TOR enjoyed fluidity above their melting point. MFR/MVR analysis on 50/50 wt.% blends conducted at 190 °C was indicative of good fluidity of GTR/LLDPE due to the linear backbone and low molecular weight of LLDPE (MFR equal to 50 g/10 min, 190 °C and 2.16 kg). Evidently, the MFI of 50/50 GTR/LLDPE was high (28.4 g/10 min), while those of 50/50 GTR/TOR, GTR/EVA, and GTR/EOC were 11.45, 2.04, and 1.01 g/10 min, respectively. For 75/25 GTR-based blends, however, completely different behavior was observed. Higher values of Mooney viscosity for 75/25 GTR/TOR (36.8) and GTR/EVA (73.6) compared to the corresponding 50/50 systems were characteristic of the rubber-like behavior of GTR. RPA measurement at 100 °C was not possible, regardless of composition, while at 190 °C (close to LLDPE melting point), 75/25 GTR/LLDPE showed higher G* and η*. For other systems, GTR/EVA and GTR/EOC showed higher values than that of GTR/TOR, perhaps because of the thicker interfacial region. Mechanical properties of 50/50 blends followed a similar trend, with GTR/LLDPE taking the minimum value. When the GTR content was increased from 50 to 75 wt.%, a similar trend was observed for all blends, except for GTR/LLDPE. Morphological analyses were in agreement with the mechanical behavior, where elongated rubber-like regions that formed at the interface were observed. DSC results showed that the glass transition temperature (T_g_) of GTR remained almost unchanged at −60 °C. In conclusion, we postulated that thermoplastics with different affinity and linearity could form shells with different thicknesses around GTR and play the role of binder or processing aid.

## Figures and Tables

**Table 1:** Characteristics of used thermoplastics (based on producers’ technical data sheets).

Item	Method	Thermoplastics			
		TOR	EVA	EOC	LLDPE
Density (g/cm^3^)	ISO 1183	0.910	0.940	0.902	0.926
Melting temperature (°C)	DSC	54	87	95	-
Vicat softening temperature (10 N) (°C)	ISO 306	-	62	80	88
MFR_190 °C, 2.16 kg_ (g/10 min)	ISO 1133	12.6	1.7	1.1	50
Mooney viscosity ML (1 + 4) 100 °C	ISO 289	<10	-	-	-
Tensile strength (MPa)	ISO 527	7.5	37 *	33	13 **
Elongation at break (%)	ISO 527	400	550 *	710	120 **

* According to ASTM D882; ** According to ASTM D 638.

**Table 2:** Sample composition, coding, and processing properties of studied samples.

Component	Standard	Sample Coding							
		GTR/TOR 50/50	GTR/EVA 50/50	GTR/EOC 50/50	GTR/LLDPE 50/50	GTR/TOR 75/25	GTR/EVA 75/25	GTR/EOC 75/25	GTR/LLDPE 75/25
GTR		50	50	50	50	75	75	75	75
TOR		50				25			
EVA			50				25		
EOC				50				25	
LLDPE					50				25
**Processing properties**									
MFR_190 °C/5 kg_ (g/10 min)	ISO 1133	11.45 ± 0.18	2.04 ± 0.03	1.01 ± 0.01	28.40 ± 0.33	-	-	-	3.61 ± 0.48
MVR_190 °C/5 kg_ (cm^3^/10 min)		12.71 ± 0.21	2.24 ± 0.03	1.14 ± 0.01	32.00 ± 0.38	-	-	-	3.71 ± 0.50
Mooney viscosity ML(1 + 4) 100 °C	ISO 289	11.6 ± 0.2	40.1 ± 3.9	-	-	34.3 ± 3.6	62.1 ± 16.3	-	-
RPA G* at 150% 100 °C (kPa)	Non-standardized method using RPA at 100 °C	6.4 ± 0.3	26.9 ± 3.1	29.3 ± 3.9	-	25.7 ± 1.6	48.5 ± 13.6	58.3 ± 11.1	-
RPA G* at 300% 100 °C (kPa)		6.2 ± 0.4	18.8 ± 3.8	20.8 ± 2.7	-	18.1 ± 2.6	30.9 ± 6.8	40.6 ± 6.9	-
RPA η* at 150% 100 °C (Pa·s)		10,188 ± 405	42,577 ± 4857	46,439 ± 6110	-	40,666 ± 2578	76,833 ± 21,495	92,446 ± 17,545	-
RPA η* at 300% 100 °C (Pa·s)		9881 ± 607	29,790 ± 6089	33,017 ± 4205	-	28,618 ± 4155	48,991 ± 10,772	64,375 ± 10,893	-
RPA G* at 150% 190 °C (kPa)	Non-standardized method using RPA at 190 °C	1.5 ± 0.1	5.3 ± 0.5	7.7 ± 0.6	0.6 ± 0.0	9.3 ± 0.3	12.0 ± 0.8	18.8 ± 0.3	5.2 ± 0.0
RPA G* at 300% 190 °C (kPa)		1.6 ± 0.0	4.6 ± 0.6	6.8 ± 0.7	0.6 ± 0.0	6.8 ± 0.0	10.0 ± 0.5	14.4 ± 0.3	3.9 ± 0.0
RPA η* at 150% 190 °C (Pa·s)		2382 ± 89	8468 ± 840	12,277 ± 958	883 ± 73	14,749 ± 474	19,017 ± 1250	29,820 ± 1608	8199 ± 29
RPA η* at 300% 190 °C (Pa·s)		2574 ± 46	7251 ± 983	10,833 ± 1067	1009 ± 61	10,850 ± 11	17,601 ± 3252	22,783 ± 540	6190 ± 6

**Table 3:** Volatile organic compounds identified by GC-MS measurement during preparation of GTR/thermoplastics blends.

Retention Time (min)	Identified Compound	Chemical Structure	Molecular Weight (g/mol)	Match Quality (%)	Source	References
4.02	benzene	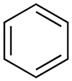	78.11	96	styrene-butadiene rubber present in GTR	[28]
5.30	toluene	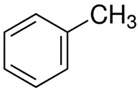	92.14	94	styrene-butadiene rubber present in GTR	[28,29]
6.60	ethylbenzene	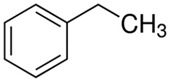	106.17	95	styrene-butadiene rubber present in GTR	[30,31]
6.73	xylene	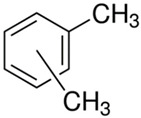	106.17	95	styrene-butadiene rubber present in GTR	[28,29]
7.03	styrene	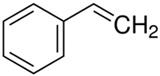	104.15	95	styrene-butadiene rubber present in GTR	[28,29]
7.91	cyclooctane	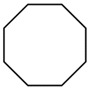	112.21	97	aliphatic thermoplastics and natural rubber present in GTR	-
8.17	benzaldehyde	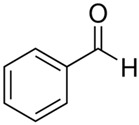	106.12	90	styrene-butadiene rubber present in GTR	[28]
8.79	α-methylstyrene	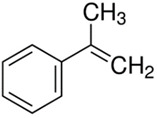	118.18	93	styrene-butadiene rubber present in GTR	[31]
9.31	decane	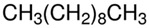	142.28	97	aliphatic thermoplastics and natural rubber present in GTR	-
9.92	limonene	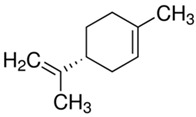	136.23	94	natural rubber present in GTR	[32,33,34]
10.21	acetophenone	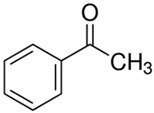	120.15	93	styrene-butadiene rubber present in GTR	-
11.22	undecane	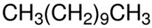	156.31	96	aliphatic thermoplastics and natural rubber present in GTR	-
12.98	dodecene	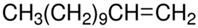	168.32	97	aliphatic thermoplastics and natural rubber present in GTR	-
13.91	hexylbenzene	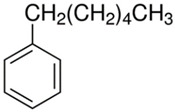	162.27	94	styrene-butadiene rubber present in GTR	-
14.58	tridecane	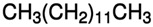	184.36	97	aliphatic thermoplastics and natural rubber present in GTR	-
16.05	tetradecane	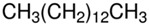	198.39	96	aliphatic thermoplastics and natural rubber present in GTR	-

**Table 4:** Physico-mechanical properties of GTR/thermoplastics blends.

Property	Sample Coding							
	GTR/TOR50/50	GTR/EVA 50/50	GTR/EOC 50/50	GTR/LLDPE 50/50	GTR/TOR 75/25	GTR/EVA 75/25	GTR/EOC 75/25	GTR/LLDPE 75/25
Tensile strength (MPa)	4.4 ± 0.3	3.4 ± 0.1	3.9 ± 0.2	5.4 ± 0.4	4.4 ± 0.2	3.4 ± 0.2	3.4 ± 0.2	2.9 ± 0.2
Elongation at break (%)	293 ± 52	329 ± 56	457 ± 97	41 ± 10	440 ± 13	436 ± 6	382 ± 13	66 ± 14
Hardness (Shore A)	82 ± 1	74 ± 1	80 ± 1	92 ± 1	69 ± 1	65 ± 1	69 ± 1	79 ± 1
Density at 20 °C (g/cm^3^)	1.001 ± 0.008	1.047 ± 0.004	1.021 ± 0.001	1.034 ± 0.002	1.086 ± 0.004	1.098 ± 0.001	1.087 ± 0.002	1.086 ± 0.007
Density at 190 °C (g/cm^3^)	0.901 ± 0.005	0.911 ± 0.002	0.882 ± 0.005	0.888 ± 0.003	- *	- *	- *	0.974 ± 0.004
Swelling degree (%)	- *	170 ± 1	80 ± 1	66 ± 2	- *	183 ± 2	120 ± 2	109 ± 2
Sol fraction (%)	- *	11.0 ± 0.1	6.6 ± 0.1	6.7 ± 0.3	- *	12.8 ± 0.3	10.0 ± 0.1	11.5 ± 0.3

* Not measurable in studied conditions.

**Table 5:** Comparison of literature data regarding GTR/thermoplastics blends.

Composition	Sample Preparation	Performance Properties	References
GTR/recycled PE 70/30	Twin-screw extrusion at 180 °C. Subsequently, injection molding at 190 °C.	Tensile strength: ~3.8 MPa * Elongation at break: ~45% *	[19]
GTR/EVA 70/30 and 80/20	High-temperature shear deformation at 160 °C. Subsequently, compression molding or injection molding	Tensile strength: 1.6–2.7 MPa Elongation at break: 100–140% Melt flow rate (no information about measurement conditions): 0.1–1.0 g/10 min	[20]
GTR/PP 80/20 and 60/40	Internal mixer at 165 °C, components mixed around 10 min. Subsequently, compression molding at 190 °C for 10 min.	Tensile strength: ~2–7 MPa * Elongation at break: ~15–38% *	[38]
GTR/HDPE 80/20	Internal mixer at 160 °C, components mixed around 15 min.	Tensile strength: ~2.2–4.3 MPa * Elongation at break: ~10–58% *	[39]
GTR/thermoplastics 75/25 and 50/50	Internal mixer at 180 °C, components mixed around 8 min.	Tensile strength: 0.4–5.4 MPa Elongation at break: 41–611% MFR_190 °C/5 kg_: 1.01–28.40 g/10 min	This study

* Tensile parameters estimated from graphs.

**Table 6:** Thermal parameters of GTR/thermoplastic blends determined by DSC.

Sample	T_m_ (°C)	ΔH_m_ (J/g)	T_g_ (°C)
GTR/TOR 50/50	59	27	−60
GTR/TOR 75/25	56	15	−60
GTR/EVA 50/50	87	30	−61
GTR/EVA 75/25	87	16	−60
GTR/EOC 50/50	96	36	−59
GTR/EOC 75/25	94	18	−59
GTR/LLDPE 50/50	124	60	−59
GTR/LLDPE 75/25	123	30	−61

## Data Availability

Not applicable.
